# Incidental Counting: Speeded Number Naming Through Finger Movements

**DOI:** 10.5334/joc.49

**Published:** 2018-11-15

**Authors:** Elena Sixtus, Oliver Lindemann, Martin H. Fischer

**Affiliations:** 1 Research Group “Motor Control and Cognition”, University of Potsdam, DE; 2 Empirical Childhood Research, University of Potsdam, DE; 3 Department of Psychology, Education and Child Studies, Erasmus University Rotterdam, NL; 4 Department of Psychology, University of Potsdam, DE

**Keywords:** embodied cognition, finger counting, numerical cognition, number naming

## Abstract

The first steps in numerical cognition are usually done in conjunction with fingers. Following the assumption that abstract concepts stay associated with the sensory-motor information that was present during their acquisition and consolidation, mental number representations should always be associated with the respective finger counting components. We tested whether finger movements that imply finger counting actually prime the corresponding number concepts in adults. All participants counted number 1 with their thumb and incremented sequentially to number 5 with their pinky. In the experiment, participants sequentially and repeatedly pressed five buttons from thumb to pinky. Each button press triggered the visual presentation of a random number between 1 and 5 that had to be named aloud, resulting in 20% counting-congruent and 80% counting-incongruent finger-number mappings. Average naming latencies were significantly shorter for congruent than incongruent finger-number combinations. Furthermore, there was a distance effect where primes partly co-activated numerically close target numbers and with decreasing priming for more distant prime-target pairs. Overall, these results provide further evidence that number representations are strongly associated with finger counting experience, making fingers an effective tool for number comprehension.

## Introduction

Hands and fingers play an important role in mental number representation, as evidenced by both neuroscientific and behavioural studies. A neuroanatomical link was identified in overlapping cerebral areas for finger and number representations (left angular gyrus, cf. Rusconi, Walsh, & Butterworth, 2005; horizontal part of the intraparietal sulcus (hIPS) and posterior superior parietal lobule (PSPL), cf. Andres, Michaux, & Pesenti, 2012; Tschentscher, Hauk, Fischer, & Pulvermüller, 2012). Also, hand muscles, but not arm and foot muscles showed increased corticospinal excitability when transcranial magnetic stimulation was applied to the primary motor cortex during counting (Andres, Seron, & Olivier, 2007). Behavioural studies provide further evidence that hands and numbers interact (Badets, Bouquet, Ric, & Pesenti, 2012; Di Luca, Granà, Semenza, Seron, & Pesenti, 2006; Imbo, Vandierendonck, & Fias, 2011; Sixtus, Fischer, & Lindemann, 2017). Imbo et al. (2011) found that moving participants’ hands during a counting task reduced their performance in a numerical task. Similarly, Michaux, Masson, Pesenti, and Andres (2013) reported that active finger movements, but not foot movements, selectively interfered with simple addition and subtraction problems (but not with simple multiplication problems which are supposedly solved through direct retrieval instead of mental calculation). Additionally, Soylu and Newman (2016) found that one-digit, but not two-digit addition problems showed interference effects from active sequential finger movements when compared to a condition with no concurrent finger movements. One possible interpretation is that early acquisition of one-digit addition skills relies more on finger counting techniques than does two-digit addition, which is why only this presumably easier task is affected by concurrent sequential finger movements. Importantly, in those three studies reported above the hand movements were external to the numerical task, thereby possibly exerting unspecific interference over associated number concepts. It is thus conceivable that number-congruent movements would facilitate number processing. The question then arises what makes a movement number-congruent.

The present study aims to investigate the cognitive consequences of number-congruent movements as defined by the congruency between fingers and numbers. The following paragraph will explain the underlying idea which relies on two pre-experimentally established associations between fingers and numbers.

The first association is based on the spatial interaction between the cognitive representation of numbers and the physical alignment of fingers. It is explained by the idea that numbers are mentally represented along a mental number line (MNL) with small numbers to the left of larger numbers (in Western cultures; Dehaene, 1992). A spatially congruent movement associates left-sided movements with small numbers and right-sided movements with large numbers. Indeed, such a facilitating effect is commonly reported, especially in the form of the so-called SNARC effect (spatial-numerical association of response codes; Dehaene, Bossini, & Giraux, 1993): left-sided responses are faster to small than large numbers and right-sided responses are faster to large than small numbers. The SNARC is thought to be independent from the hands or properties of the body because responses given with crossed hands show the same spatial congruency effect (Dehaene et al., 1993; but see Wood, Nuerk, & Willmes, 2006). This effect was also reported within one hand: fingers which were located further to the left were associated to smaller numbers and fingers located further to the right were associated to larger numbers, regardless of finger identity (Brozzoli et al., 2008).

The second association between fingers and numbers is embodied: numbers become embodied through habitual finger counting where each of the successively extended (or folded, depending on finger counting habits) fingers is consistently used in conjunction with a specific number (name), thus establishing the association of interest. A study by Di Luca et al. (2006) supports this claim: participants responded to numbers with various finger-to-number mappings; responses were fastest when the mapping conformed to the participants’ finger counting habits – faster also than with a spatial, MNL-conforming (i.e., increasing left-to-right) mapping.

Extending this evidence, Riello and Rusconi (2011) reported a co-existence of both spatial and embodied effects. In their study, participants responded to numbers with button presses of the index and middle fingers in four separate conditions, using either their left or right hand with the palms either up or down. All participants indicated to count with number sizes increasing from thumb to pinky. Thus, for the left hand in a palm up-position and for the right hand in a palm down-position, MNL- and finger counting directions coincided. Intriguingly, it was only for those response configurations that a SNARC effect was present. In the other configurations the congruency benefit of interest was seemingly cancelled out by the competing spatial and embodied number representations.

The two studies reported above show that active finger involvement influences mental number processing. Additionally, there is evidence for effects of passive finger involvement on mental number processing (e.g., Di Luca & Pesenti, 2008; Sixtus, Lindemann, & Fischer, 2018). As we have shown previously, tactile finger stimulations pre-activated associated mental number concepts (Sixtus et al., 2018). Di Luca and Pesenti (2008) furthermore reported that passive subliminal visual presentation of finger montring postures (i.e., finger configurations that are used to show numbers without counting up to that number first) primed mental number processing. Moreover, in a priming paradigm with (supraliminal) visual presentation of finger montring postures of numbers 1 to 5 as primes and with numbers in Arabic or verbal format as targets Di Luca, Lefèvre, and Pesenti (2010) found a distance effect: target naming latencies increased with increasing distance between target and prime number. When primes consisted of non-canonical postures, that is, postures that are usually not used to represent numbers, this distance effect only appeared when the target number was larger than the prime number. That is, in contrast to canonical montring postures, non-canonical postures apparently also primed all smaller numbers to a similar degree, indicating reliance on a summative representation (cf. Roggeman, Verguts, & Fias, 2007). Interestingly, Sixtus et al. (2017) found that the active production of finger counting postures had a greater impact on mental number processing than the passive (supraliminal) visual perception of the same finger postures, which emphasises the special role of active finger involvement for the association between fingers and numbers.

In the present study, we extend previous work in several ways. Participants pressed buttons with their fingers while their hand was oriented palm down and named subsequently appearing numbers. By avoiding explicit finger-number assignments (cf. Di Luca et al., 2006) and testing whether even such implicit finger-number associations were capable of priming number representations, we further explored the nature of embodied number representations. If explicit finger-number mappings were required in order to obtain measurable priming effects, this would merely indicate facilitated recall of mapping rules due to familiarity with finger counting. On the other hand, if finger-number associations are measurable implicitly, then this indicates a direct link between specific mental finger and number representations.

Consistent with the above literature, we hypothesised that finger movements should co-activate only counting-congruent embodied number representations (comparable with Di Luca et al., 2006) so that counting-congruent finger-number pairs should elicit faster verbal responses than incongruent ones (see Sixtus et al., 2018 for a similar finding for passive tactile finger stimulation) and that the priming effect should decrease with numerical distance between prime and target numbers (as Di Luca et al., 2010 found for visual perception of montring postures).^1^ However, if the spatial-numerical association was of equal strength as the embodied association between fingers and numbers, then the anticipated motor priming effect should only appear for the right hand where the embodied and spatial finger-number associations coincide when this hand is palm-down (cf. Riello & Rusconi, 2011). Importantly, we also assessed finger counting habits and tested whether a congruency effect depended on the starting hand in finger counting, that is, the hand which was spontaneously used for numbers one to five which were part of the present experiment.

## Method

The experiment consisted of a number naming task in which participants sequentially pressed buttons with the fingers of one hand, thereby triggering the display of Arabic numerals on the computer screen. Buttons were pressed such that the order of the fingers pressing the buttons conformed to usual Western finger counting habits – ascending from the thumb to pinky.

### Participants

Thirty students from the University of Potsdam took part in the experiment for course credit or money. All subjects gave written informed consent and the experiment was conducted in accordance with the ethical standards expressed in the Declaration of Helsinki. All but four participants counted from one to ten on their thumb to pinky by starting on the thumb of one hand and ending on the pinky of the other hand. One counted from thumb to pinky of the right hand twice. The remaining three participants did not reveal a consistent one-to-one correspondence of fingers to numbers and were thus excluded from congruency analyses. The final sample involved 27 participants and was composed of 26 German native speakers and one English native speaker (19 female, 8 male, mean age = 23.56 years, *SD* = 4.53). Twenty-three were self-reportedly right-handers and four were left-handers.

### Apparatus and experimental set-up

Participants were seated at a table with a computer monitor (60 cm screen diagonal; 60 Hz refresh rate) and a standard keyboard. The keyboard save five buttons (‘f’, ‘g’, ‘h’, ‘j’, and space) was covered by a sheet of paper and the uncovered buttons were centred in front of the participant. Verbal responses were spoken into a microphone (‘t.bone EM-9900’; Thomann GmbH, Burgebrach, Germany) that was connected to an audio interface (‘U46 XL’; ESI Audiotechnik GmbH, Leonberg, Germany). They were detected by a voice key device (‘SV-1 Voice Key’; Cedrus Corporation, San Pedro, USA) connected to the audio interface’s phones output. A custom-made program based on the free Python library *Expyriment* (Krause & Lindemann, 2014) controlled stimulus presentation and recording of voice onset times.

### Stimuli

Target stimuli consisted of Arabic digits 1 to 5 (text size = 35 pixels, sans serif font, light grey colour on a black background) that were positioned at the centre of the computer screen.

### Procedure

Participants were instructed to put each finger of one hand on the respective button of the computer keyboard in front of them. Instructions on the computer screen informed them whether the left or right hand was to be used in the following block (order balanced over participants). Starting with the thumb, the fingers had to press down buttons on the keyboard consecutively in the order [thumb – index finger – middle finger – ring finger – pinky]. Directly following each button press a number appeared on the screen that had to be read out aloud as quickly as possible in the participant’s native language. Reaction times (RTs) were measured from the appearance of the number until the verbal response was detected by the voice key device. The number disappeared as soon as a response was detected. After a random delay between 250 and 500 ms a fixation dot appeared at the centre of the screen. As soon as the dot had appeared, the participant could press the next button in the sequence with the appropriate finger to evoke the next number. After every 50 trials there was a short pause. In the middle of the experiment participants were instructed to change hands.

A short message appeared on the computer screen whenever an incorrect finger was used, when it took too long to press the next button (when the dot was presented for over 1500 ms) or when the next button was pressed before a voice response was detected. A message then also informed about which finger had to be used next.

The experimenter monitored the entire experiment and noted trial numbers for wrong responses or false voice onset registrations, due to external noise or the participant using a weak voice.

Finger counting habits were assessed with two methods for each participant, a number-based and a syllable-based method. The number method consisted of asking participants to “Show me how you count from one to ten with your hands”. The syllable method consisted of asking participants: “How many syllables does the children’s song ‘Alle meine Entchen’ [popular German children’s song] have until the word ‘See’?” (correct response: 11). Participants were encouraged to use their hands in the few cases where they did not do it spontaneously. The English speaking participant, who did not know the song, was instead asked about the number of syllables of the first verse of the song “What shall we do with a drunken sailor” (correct response: 10). In all cases the starting hand and finger counting sequence were recorded. The order of assessments, which took place before or after the experiment, was counterbalanced across participants.

### Design

Participants completed 500 experimental trials in total: 2 hands × 5 fingers × 5 numbers (1–5) × 10 repetitions. A short training of 20 trials (or more if required) was inserted at the beginning and when changing hands. Order of hands was counterbalanced across participants. Target numbers were distributed quasi-randomly with the only restriction that the same number could not turn up more than twice in a row. The entire experiment took approximately 30 minutes.

## Results

Raw data and the analysis script are available online via https://osf.io/nur86. Trials where participants pressed a wrong button (0.58%) or failed to press the next button within 1500 ms (0.30%) were eliminated from the data. Note that these trials were immediately repeated in the experiment. Further error trials (that were not repeated) were excluded from analyses: errors consisted of incorrect verbal responses (0.30%), false voice onset registrations (2.74%), and missed verbal responses (0.72%), excluding 3.75% of the trials. Then, trials with extreme RT values, which were presumably attributable to a lack of concentration, were eliminated (RT < 250 ms or > 1500 ms; 0.33%).

Next, results from the two methods of assessing the starting hand were compared. Table 1 lists the number of participants who started counting on the left or right hand under each method. Although 33% of the participants had nonmatching starting hands in the two counting tasks, McNemar‘s exact test revealed no evidence for a statistically significant difference between the results from the two methods of assessment, *p* = .344. However, it has to be noted that statistical power for this test is insufficient to reject the hypothesis of the presence of a difference, 1-β = 0.23 (G*Power; Faul, Erdfelder, Lang, & Buchner, 2007).

**Table 1:** Number of participants with left/right starting hand in the two counting tasks.

		Starting hand for syllables		Total

		Left	Right	

**Starting hand for numbers**	**Left**	5	3	8
	**Right**	7	15	22

**Total**		12	18	30

*Note.* Five participants were left-handers. One of them started both syllable and number counting with the left hand, two started only syllable counting with the left hand, two started only number counting with the left hand, and none started both with the right hand.

To account for different verbal onset times for the different number words, RTs were adjusted regarding mean RTs per target number. Specifically, first mean RTs were calculated for each participant and response (mean RTs: number 1: 646 ms, *SD* = 82 ms, number 2: 733 ms, *SD* = 77 ms, number 3: 671 ms, *SD* = 78 ms, number 4: 705 ms, *SD* = 82 ms, number 5: 669 ms, *SD* = 74 ms). Second, mean RTs for each participant (averaged over all numbers) were calculated. The differences between these two values constitute the specific effects of each number word for each participant. Finally, these difference values were subtracted from every RT in the experimental trials that came from the corresponding participant and target number to adjust for the above-mentioned effect of the number words. Note that this adjustment does not affect the congruency analyses, but the subsequent analysis of the distance effect.

**Figure 1:**
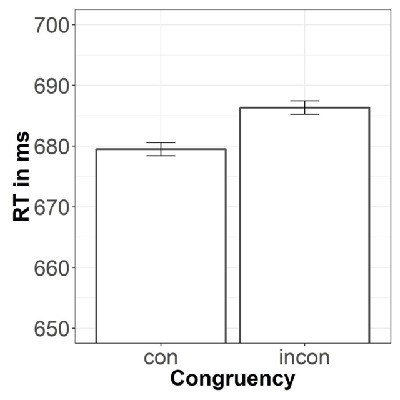
Reaction times in ms for congruent and incongruent finger-number pairs. Error bars represent within-subject standard errors as suggested by Cousineau (2005).

For the main analysis regarding the congruency effect, RTs were averaged per subject, hand, finger, and target number. Congruent trials included finger-number pairs thumb-1; index finger-2; middle finger-3; ring finger-4; and pinky-5. All other trials were defined as incongruent. The 2 × 2 ANOVA thus included the within-subject factors Congruency (congruent/incongruent) and Hand (left/right). There was a significant main effect of Congruency, *F*(1, 26) = 9.80, *p* = .004, η^2^_p_ = .27. RTs for congruent trials (680 ms, *SD* = 79 ms) were shorter than for incongruent trials (686 ms, *SD* = 80 ms; see Figure 1). There was no significant main effect of Hand, *F*(1, 26) = 2.26, *p* = .145, and no significant interaction of Congruency and Hand, *F*(1, 26) < 1.

In addition, we performed two further ANOVAs, each including one of the measures of the starting hand in finger counting, to find whether one (or both) of them captured a finger counting habit which influenced the association between fingers and numbers. The first 2 × 2 × 2 ANOVA included the same within-subject factors as before and the between-subject factor Starting hand numbers (left/right). There again was a significant main effect of Congruency, *F*(1, 25) = 5.71, *p* = .025, η^2^_p_ = .19. There were no significant main effects of Hand, *F*(1, 25) = 2.14, *p* = .156, Starting hand numbers, *F*(1, 25) = 2.73, *p* = .111, and no significant interaction effects, all *F*s < 1.

The second 2 × 2 × 2 ANOVA included the same within-subject factors as before and the between-subject factor Starting hand syllables (left/right). There again was a significant main effect of Congruency, *F*(1, 25) = 10.72, *p* = .003, η^2^_p_ = .30. There were no significant main effects of Hand, *F*(1, 25) = 2.07, *p* = .162, Starting hand syllables, *F*(1, 25) < 1, and no significant interaction effects, all *p*s > .231.^2^

**Figure 2:**
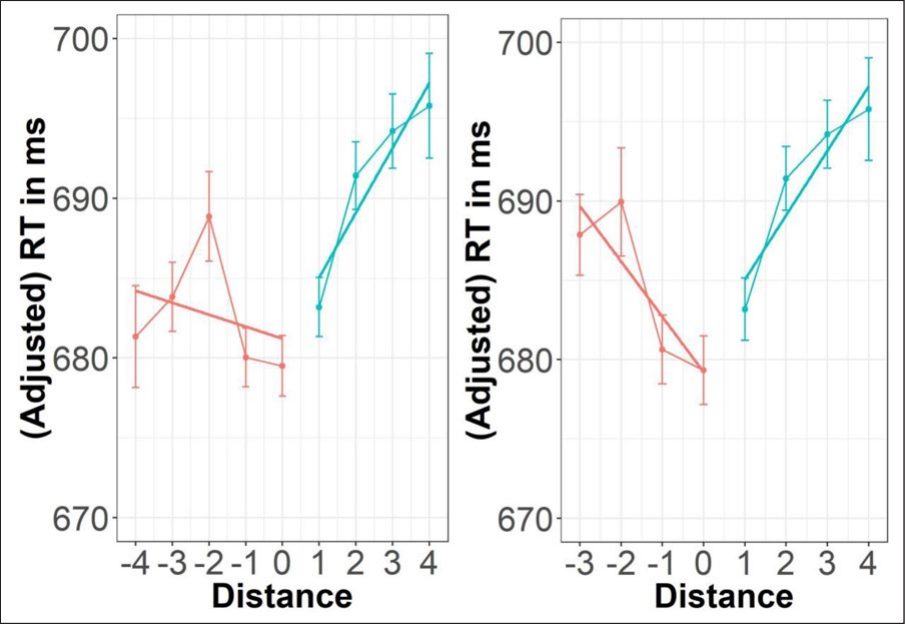
Adjusted reaction times in ms for each prime-target distance. Left: including all prime numbers; right: excluding prime number 5 (pinky) to match Di Luca et al.’s (2010) analysis. Error bars represent within-subject standard errors, as suggested by Cousineau (2005).

Finally, a prime-target distance effect was investigated.^3^ The numerical distance was calculated as the difference between the number associated with the moved finger (henceforth labelled prime number) and the target number. That is, for negative values the prime number was larger than the target number (see Figure 2) and a distance of zero corresponds to congruent trials. We conducted a linear mixed model analysis using R package lme4 (Bates, Mächler, Bolker, & Walker, 2015) with the fixed factors Distance and Side and the random factor Participant. The continuous factor Distance was defined as the absolute value of the numerical distance indicated above. The categorical factor Side indicated whether the target number was smaller than or equal to the prime number (left side) versus larger than the prime number (right side; see Figure 2; cf. Di Luca et al., 2010 for a similar approach). *t*-values larger than 2 were considered as statistically significant for the linear mixed models (Baayen, 2008). RTs significantly increased with increasing distance and the Distance × Side interaction revealed that this increase was significantly stronger for the right side than for the left side (see Table 2 and Figure 2, left panel). Note that Di Luca et al. (2010) restricted their analysis to prime numbers 1 to 4. Importantly, when restricting the present data in the same way, the significant interaction effect disappeared (see Table 2 and Figure 2, right panel).

**Table 2:** Results of the linear mixed-effects analysis. Left: including all prime numbers; right: excluding prime number 5.

Effect (fixed)	All prime numbers			Excluding prime number 5		

	β	*SE*	*t*	β	*SE*	*t*

Distance	3.13	0.71	4.39	4.17	0.86	4.86
Side	0.23	1.51	0.16	–0.28	1.61	–0.17
Distance × Side	–1.56	0.71	–2.19	–0.53	0.86	–0.62

## Discussion

The main objective of the present study was to investigate whether and how finger movements affected number processing. We focused on embodied number representations, that is, pre-experimentally established finger-number associations that comply with finger counting habits. Repeatedly performing finger movements in this habitual order resulted in speeded naming latencies for counting-congruent finger-number pairs. The underlying mechanism presumably involved priming of the associated number concepts through finger movements. This interpretation is supported by the finding that there was a significant distance effect, where RTs increased with an increasing distance of the target from the prime number. The present results complement previous findings of passive finger stimulations activating associated number concepts (Sixtus et al., 2018) and support embodied accounts of cognition according to which all knowledge, even supposedly ‘abstract’ numerical knowledge, remains associated with the sensory and motor activation that was present during its acquisition (Barsalou, 2008; Fischer, 2012; Fischer & Coello, 2016; Pulvermüller, 2013).

Importantly, the congruency effect in our task did not depend on the starting hand in finger counting, or on the active hand during the experiment, or on their interaction. Interactions with the starting hand would have been expected if the starting hand were an intra-individually stable trait. Interactions with the active hand were predicted from the spatial associative account according to which the congruency benefit is limited to those conditions where fingers are spatially oriented in alignment with the left-right increasing MNL. We discuss these additional observations and their implications in turn.

Regarding the starting hand, it might have been expected that the effect only appear in the hand spontaneously used for numbers one to five which were part of the present experiment. Previous findings have been partly inconsistent regarding the impact of the individually preferred starting hand. While Fischer (2008) found a SNARC effect only for left-starters, Fabbri (2013) reported a SNARC effect only for right-starters. The study of Newman and Soylu (2014) suggested that right-starters outperformed left-starters in mental addition and Tschentscher et al. (2012) showed that left- and right-starters differed in their specific neuronal representation of small numbers, that is, whether the right or left premotor and motor cortex were specifically active for processing small numbers (see also Sato, Cattaneo, Rizzolatti, & Gallese, 2007). However, the finding of the present study that one third of the participants had nonmatching starting hands in the two counting tasks suggests that both hands can be assigned to small or large numbers (one to five or six to ten, respectively) rather flexibly (see also Wasner et al., 2014a, 2014b). This is in line with previous findings that priming through finger postures does not depend on the individually preferred starting hand (Sixtus et al., 2017, Experiment 2). Due to this flexibility, both hands presumably habitually represent both small and large numbers, resulting in measurable associations between the investigated small numbers and fingers of both hands. The divergent results from the two different measures of starting hand are worth further consideration. Wasner et al. (2014a, 2014b) already reported flexible finger-number mappings and showed that finger counting behaviour is influenced by situational conditions like for example the visual availability of the own hands and the spontaneity of the finger counting process; however, they used different participants for related tasks that all included counting with the fingers (Wasner et al., 2014a) or they used very different tasks for the same participants, whereof only one consisted in counting with the fingers (Wasner et al., 2014b). The present results further emphasise that the starting hand in finger counting is strongly affected by situational factors: one third of the participants were inconsistent in their starting hand with only about half an hour lying between the two measures and with the only difference being the items to be counted. That is, the choice of starting hand is situated in the sense that it does not merely depend on intra-individually stable habits, but takes into account the situational condition. The situational condition, in the case of the present study, was primarily given by the items that were required to be counted. However, it remains to be investigated whether the divergent results reported within this study prove to become statistically significant in larger samples. If this is the case, it furthermore remains to be investigated whether the choice of starting hand is stable within each item category (i.e., numbers or syllables) and which other situational factors – in addition to item category as well as visual availability of the own hands and the spontaneity of the finger counting process (cf. Wasner et al., 2014a) – might have significant effects.

Regarding the active hand, it might have been expected that the congruency effect would be stronger on the right than left hand because only for the right hand did the embodied finger counting direction coincide with the spatial-numerical association imposed by the direction of the MNL, that is, smaller numbers on the left and bigger numbers on the right. The congruency effect for the left hand appears to conflict with Riello and Rusconi’s (2011) results where finger counting direction needed to be spatially aligned with the MNL for the congruency effect to appear. However, the two studies differ in their definitions of congruency: Riello and Rusconi (2011) measured the SNARC effect, looking for a more general space-magnitude congruency, and thereby coded whole magnitude dimensions (smaller or larger than five) to single fingers, while we coded discrete finger-number congruency, that is, one number per finger. This indicates that, while general magnitude processing may depend on finger-space alignment, specific finger-number associations do not.

Moreover, Riello and Rusconi (2011) found a reliable spatial-numerical association only in the parity task while it “fell (…) far from significance in the magnitude task [4 ms; *F*(1, 46) =1.131, *p* > 0.10]” (p. 6). This aspect of their results indicates that the level of number processing might be important, with better sensitivity for implicit compared to explicit magnitude processing (cf. Shaki & Fischer, 2018). Arguably, our present naming task imposed even less semantic number knowledge retrieval compared to a parity task, thus increasing sensitivity of the measurement.

The present study complements the work of Di Luca et al. (2006) by revealing the bi-directional relationship between finger movements and mental number representations: in Di Luca et al.’s (2006) experiment, the number was given and responses were finger movements while in the present experiment, finger movements were given and responses were vocalised number names. Furthermore, the present study reveals the implicit link between numbers and fingers: in their experiment the task explicitly assigned numbers to fingers, which was not the case in the present experiment where numbers appeared quasi-randomly with only 20% finger-number congruent trials scattered throughout the experiment.

The present study furthermore complements the work of Di Luca et al. (2010) by revealing that also active finger movements as primes yield a distance effect. However, there were diverging results regarding the interaction of absolute distance and the factor Side (i.e., target numbers smaller than or equal to prime numbers versus target numbers larger than prime numbers) when including versus excluding prime number 5 (i.e., trials involving the pinky). When analysing the reduced prime set which also Di Luca et al. (2010) had analysed, the resulting slopes closely resembled their slopes for their canonical primes. That is, performing sequential finger movements and perceiving pictures of finger montring configurations apparently co-activates number concepts in a similar fashion.

One concern with the present experiment might be a potential subvocalisation of the fingers’ associated numbers, which would indicate a strong association between fingers and their related number names, but not necessarily between fingers and number concepts beyond their names. However, participants had to actively pronounce mostly (i.e., in 80% of the cases) unassociated number names, so that we are confident that potential subvocalisation strategies were interrupted by this task.

A limitation of the present study is that fingers were only moved in a sequence that matched finger counting. This leaves the possibility that the effect did not emerge through finger-number associations, but through a sequence effect independent from finger identity. We tried to minimise such an effect by having always 50 uninterrupted trials per mini-block so that the thumb was not only moved as first finger but also as sixth, eleventh etc., the index not only as second finger but also as seventh, twelfth etc.… It is nevertheless still possible that the leap from the pinky back to the thumb induced a resetting of participants’ internal counter to ‘one’, which could have been induced by the spatial leap and not by finger identity. With this study alone we can therefore not conclusively distinguish between an effect of finger-number associations and a sequence effect. However, there are other studies pointing towards finger-number associations without the fingers necessarily being involved in an ordered sequence: when a tactile device stimulated participants on the index, middle, or ring finger two, three, or four times, their responses regarding the amount of stimulations were more accurate and faster on the counting-congruent fingers (Sixtus et al., 2018). The present study extends this finding by showing that also actively produced finger movements prime number concepts. A follow-up study could set out to replicate the present results using another fixed sequence or even random finger movements instead of the counting-congruent sequence.

In conclusion, the present study provides further evidence that number representations are strongly associated with finger counting experience. This persistent association indicates that fingers are an effective tool in the acquisition of number concepts, which creates a lifelong link between the mental representation of fingers and numbers.

## Data Availability

The raw data and analysis script are available via the Open Science Framework platform at https://osf.io/nur86.

## Footnotes

Finger-number congruency can be defined as following from counting or montring experience; in counting each finger is habitually used for one specific number; it focuses on the ordinal aspect of numbers. In montring certain finger configurations are habitually used for specific numbers; it focuses on the cardinal aspect of numbers (i.e., the fact that a number can express a set size). Montring configurations differ from counting configurations in some individuals: in a German sample the majority of individuals had matching counting and montring configurations for all numbers from 1 to 5, except 4 (Wasner, Moeller, Fischer, & Nuerk, 2014b). However, we were primarily interested in specific finger-number associations and therefore decided to focus on the ordinal aspect of numbers which arises from finger counting experience. Moreover, the nature of the task (i.e., sequential finger movements) presumably activates associations established by finger counting.

All results were statistically identical whether or not the data of the English native speaker were included and we report the full data.

We would like to thank an anonymous reviewer for suggesting the analysis of the distance effect.
